# Study on the Mechanical Properties of Optimal Water-Containing Basalt Fiber-Reinforced Concrete Under Triaxial Stress Conditions

**DOI:** 10.3390/ma18143358

**Published:** 2025-07-17

**Authors:** Kaide Liu, Songxin Zhao, Yaru Guo, Wenping Yue, Chaowei Sun, Yu Xia, Qiyu Wang, Xinping Wang

**Affiliations:** Shaanxi Key Laboratory of Safety and Durability of Concrete Structures, Xijing University, Xi’an 710123, China; 13333477600@163.com (Y.G.); 20180202@xijing.edu.cn (W.Y.); chao_wei_106@126.com (C.S.); 17607139575@163.com (Y.X.); realwanglaoji@163.com (Q.W.); 15835086038@163.com (X.W.)

**Keywords:** basalt fiber-reinforced concrete, NMR, microstructural optimization, triaxial compression, failure characteristics, mechanical properties

## Abstract

In response to the high-performance requirements of concrete materials under complex triaxial stress states and water-containing environments in marine engineering, this study focuses on water-containing basalt fiber-reinforced concrete (BFRC). Uniaxial compression and splitting tensile tests were conducted on specimens with different fiber contents (0.0%, 0.05%, 0.10%, 0.15%, and 0.20%) to determine the optimal fiber content of 0.1%. The compressive strength of the concrete with this fiber content increased by 13.5% compared to the control group without fiber, reaching 36.90 MPa, while the tensile strength increased by 15.9%, reaching 2.33 MPa. Subsequently, NMR and SEM techniques were employed to analyze the internal pore structure and micro-morphology of BFRC. It was found that an appropriate amount of basalt fiber (content of 0.1%) can optimize the pore structure and form a reticular three-dimensional structure. The pore grading was also improved, with the total porosity decreasing from 7.48% to 7.43%, the proportion of harmless pores increasing from 4.03% to 4.87%, and the proportion of harmful pores decreasing from 1.67% to 1.42%, thereby significantly enhancing the strength of the concrete. Further triaxial compression tests were conducted to investigate the mechanical properties of BFRC under different confining pressures (0, 3, and 6 MPa) and water contents (0%, 1%, 2%, and 4.16%). The results showed that the stress–strain curves primarily underwent four stages: initial crack compaction, elastic deformation, yielding, and failure. In terms of mechanical properties, when the confining pressure increased from 0 MPa to 6 MPa, taking dry sandstone as an example, the peak stress increased by 54.0%, the elastic modulus increased by 15.7%, the peak strain increased by 37.0%, and the peak volumetric strain increased by 80.0%. In contrast, when the water content increased from 0% to 4.16%, taking a confining pressure of 0 MPa as an example, the peak stress decreased by 27.4%, the elastic modulus decreased by 43.2%, the peak strain decreased by 59.3%, and the peak volumetric strain decreased by 106.7%. Regarding failure characteristics, the failure mode shifted from longitudinal splitting under no confining pressure to diagonal shear under confining pressure. Moreover, as the confining pressure increased, the degree of failure became more severe, with more extensive cracks. However, when the water content increased, the failure degree was relatively mild, but it gradually worsened with further increases in water content. Based on the CDP model, a numerical model for simulating the triaxial compression behavior of BFRC was developed. The simulation results exhibited strong consistency with the experimental data, thereby validating the accuracy and applicability of the model.

## 1. Introduction

With the continuous advancement of national efforts in the development and utilization of marine resources, the construction of marine engineering infrastructure has been rapidly growing, among which large-scale undersea projects such as cross-sea tunnels have become an important part of marine development [1,2,3]. The stress conditions of these undersea structures are highly complex and predominantly characterized by triaxial stress states [4,5]. Against this backdrop, concrete has been widely used in undersea construction due to its high strength, excellent impermeability, and corrosion resistance [6,7]. However, as a brittle material, concrete is prone to cracking and has poor toughness, which necessitates the incorporation of fibrous materials to enhance its performance [8,9,10]. Basalt fiber (BF), with its high strength, excellent heat resistance, acid and alkali corrosion resistance, and environmental friendliness, has gradually emerged as an ideal material for concrete modification [11,12,13]. The addition of an appropriate amount of basalt fiber can significantly improve the mechanical properties, durability, and crack resistance of concrete, effectively reducing the propagation of internal cracks and enhancing its freeze–thaw and impact resistance [14,15]. Therefore, it is of great significance to investigate the mechanical properties of basalt fiber-reinforced concrete with optimal fiber content under triaxial stress conditions.

Currently, research on basalt fiber-reinforced concrete (BFRC) has yielded rich results. Wang et al. [16] conducted large eccentric compression tests on conventional concrete and BFRC components, finding that compared with conventional concrete components, the ultimate bearing capacity of BFRC components increased by 30.3%, the cracking load significantly increased by 42.9%, and the enhanced loading characteristics of delayed crack initiation, increased number of cracks, and reduced maximum crack width were observed. Li et al. [17] investigated the properties of ordinary and BFRC through staged fatigue loading tests. The results showed that the average pore size of BFRC decreased by 16.39–21.06% compared with ordinary concrete, and porosity and total pore volume were highly correlated with flexural strength. It was also found that fibers significantly improved the pore structure. Qin et al. [18] demonstrated that the incorporation of basalt fibers significantly improved the mechanical properties of concrete. The bridging effect of fibers improved the pore structure, reduced the number of microcracks, and inhibited their propagation, thereby enhancing the density and overall performance of concrete. Wang et al. [19] revealed that basalt fibers inhibited crack propagation and reduced crack width and number through a bridging effect, thereby enhancing the toughness and integrity of concrete while improving its density and mechanical properties. Xie et al. [20] found that basalt fibers significantly enhanced the dynamic splitting tensile properties of concrete and improved the integrity after failure. Fibers inhibited the development of shear failure zones and altered crack propagation patterns to exert crack resistance. Under low strain rates, pullout failure was observed, while under high strain rates, pull-apart failure occurred. Liu et al. [21] investigated the mechanical properties of BFRC with different fiber volume fractions through SHPB experiments. It was found that fibers significantly increased the compressive and flexural strength of concrete and optimized the pore structure, with the most significant performance improvement at a volume fraction of 0.3%. Li et al. [22] studied the mechanical properties of short-cut basalt fiber concrete and found that the tensile and compressive strength of specimens first increased and then decreased with increasing fiber content, reaching a peak at a fiber content of 0.3%. Xu et al. [23] conducted static compression tests on salt-frozen BFRC and found that fiber incorporation could slow down the rate of mechanical property degradation under salt freezing, with the best anti-salt-freezing effect at a fiber content of 0.15%. Jia et al. [24] conducted uniaxial compression tests on BFRC specimens in dry and saturated states and found that the peak stress of saturated specimens was lower than that of dry specimens. Wang et al. [25] performed uniaxial compression tests on BFRC specimens cured at different relative humidity levels (35%, 55%, 75%, and 95%). With increasing curing relative humidity, the average strain rate of the specimens decreased, the peak stress increased, the energy dissipation capacity enhanced, the degree of fragmentation reduced, and the fractal dimension decreased. Zhao et al. [26] conducted uniaxial compression tests on BFRC and found that the compressive strength of single-mixed BFRC increased with fiber content, but the improvement was limited. Chen et al. [27] used CT scanning to study the pore structure of BFRC and found that fiber incorporation could change the pore size distribution ratio, reduce porosity, and improve the integrity of the specimens. Jia et al. [28] investigated the internal pore structure of BFRC using nuclear magnetic resonance and electron microscopy and analyzed the splitting tensile mechanical properties using a universal testing machine and split Hopkinson pressure bar. The results showed that fiber incorporation effectively reduced the porosity of concrete, enhanced its tensile strength, and reduced the ultimate strain. The good bonding between fibers and the concrete matrix effectively carried the load and inhibited crack propagation, exhibiting a strain rate strengthening effect. Meng et al. [29,30] conducted triaxial cyclic loading tests on rock samples under different confining pressures using an MTS815 testing machine (MTS Systems Corporation, Eden Prairie, MN, USA) to explore the influence of confining pressure on the energy evolution characteristics of rock samples. The results showed that the energy characteristic density of rock samples was positively correlated with confining pressure, which inhibited the dissipation and release of energy during sample failure, leading to incomplete release of elastic energy. De Maio et al. [31] introduced a novel numerical framework that integrates dynamic meshing techniques with an adaptive cohesive zone model to simulate crack initiation and propagation in quasi-brittle materials with heterogeneous microstructures. Chi et al. [32], based on the background of true triaxial compression tests on 75 cubic specimens, developed a plasticity-based constitutive model for hybrid steel–polypropylene fiber-reinforced concrete (HFRC) to accurately predict the strength and deformation characteristics of HFRC under various loading conditions.

Although current research on BFRC has made progress in aspects such as fiber content, microstructure, mechanical properties, and numerical simulation, most studies are limited to isolated analyses of microstructure, mechanical performance testing, or numerical modeling, failing to integrate these elements into a systematic and comprehensive investigation. Moreover, in-depth analysis of the mechanical properties under the combined effects of confining pressure and water content is still insufficient. Given this, the present study focuses on the mechanical properties of BFRC with optimal fiber content under triaxial stress conditions and systematically conducts the following work: First, BFRC specimens with different fiber contents were prepared, and the optimal fiber content was determined through uniaxial compression and splitting tensile tests. Second, the internal pore structure and micro-morphology of BFRC were deeply analyzed using nuclear magnetic resonance and scanning electron microscopy to reveal the optimization mechanism of fibers on the internal structure of concrete. Furthermore, triaxial compression tests were conducted to comprehensively investigate the mechanical properties of BFRC under various confining pressures and moisture contents, including stress–strain behavior, failure modes, elastic modulus, and peak strength. The results reveal the influence of confining pressure and moisture content on mechanical performance. Finally, a triaxial compression numerical model for BFRC was established based on the CDP model, aiming to provide a solid theoretical foundation for the design and application of concrete structures in complex environments such as marine engineering.

## 2. Test Preparation

### 2.1. Raw Materials and Mix Proportion of Test Pieces

**Figure 1:**
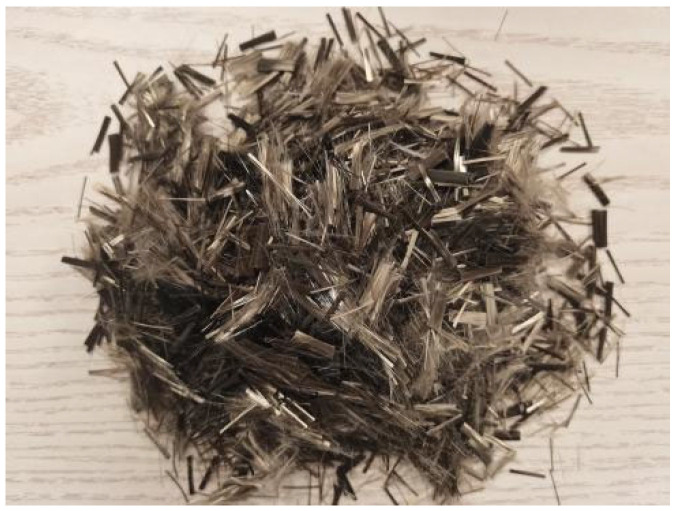
Basalt fiber.

The design strength grade of basalt fiber-reinforced concrete (BFRC) is C30. The raw materials are selected as follows: ① P.O 42.5 ordinary Portland cement; ② coarse aggregate: gravel with a particle size of 5–20 mm; ③ fine aggregate: medium sand from the Huainan Huaihe River; ④ admixture: Grade II fly ash; ⑤ fiber material: basalt fiber (Figure 1), with a length of 6 mm, a single filament diameter of 15 μm, a density of 2.63–2.65 g/cm^3^, a tensile strength of 3000–4800 MPa, an elastic modulus of 91–110 GPa, and an ultimate elongation of 3.1%; ⑥ additives: retarder and air-entraining agent produced by Chang’an Yucai; ⑦ water: ordinary tap water. According to Reference [33], the mix proportion of BFRC specimens is obtained (Table 1). In this experiment, the basic mix proportion of concrete is not changed, and the fiber is added externally by a mass fraction. For example, BFRC-0.00 in the table represents the BFRC specimen with a basalt fiber content of 0.0%, and the same applies to others.

### 2.2. Fabrication and Design of Optimal Basalt Fiber Content Specimens

#### 2.2.1. Procedure for Determining the Optimal Fiber Content Specimens

**Figure 2:**
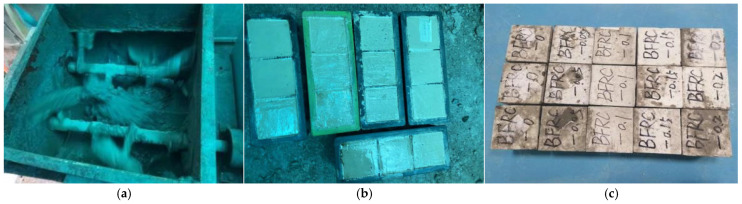
Specimen fabrication process: (**a**) mixing concrete; (**b**) vibrated and leveled specimen; and (**c**) specimen for testing.

The specific procedure for specimen fabrication is as follows: ① Preparation of Materials: Before the fabrication of concrete specimens, gravel should be screened to remove larger particles and then washed and air-dried. Natural fibers and cement should be stored in a sealed condition to prevent moisture absorption. ② Mixing Procedure: The pre-mixing method is employed for material addition and mixing. After starting the mixer, gravel and sand are first added into the mixing drum. Basalt fibers are then uniformly introduced into the mixer while it is running, ensuring that the fibers are well dispersed during the mixing process. The interaction between the gravel and fibers helps to prevent fiber agglomeration. After mixing for 2 min with the fibers, cement is added and dry-mixed for 1 min. Water is subsequently added gradually to ensure effective dissolution of the admixtures. The mixing continues for another 2 min before the mixture is discharged (Figure 2a). ③ Molding and Compaction: The mold (150 mm × 150 mm × 150 mm) is placed on a vibrating table. The concrete mixture is poured into the mold in layers, with each layer being compacted by vibration. The mold is continuously filled and leveled during the vibration process to compensate for any settlement. Once the concrete is fully compacted and no significant air bubbles or bleed water appear on the surface, the surface is smoothed with a trowel. After allowing the specimen to rest for 30 min on a flat surface, it is re-leveled and covered with plastic film to prevent rapid evaporation of moisture, which could lead to uneven shrinkage of the concrete. The specimen is then left to cure for 24 h before demolding (Figure 2b). ④ Curing: The specimens are cured in a curing chamber with a temperature of 20 ± 2 °C and a relative humidity of over 95% for 28 days. After curing, the specimens are left to air-dry outdoors for at least 6 h before testing (Figure 2c).

#### 2.2.2. Equipment for Determining the Optimal Fiber Content Specimens

**Figure 3:**
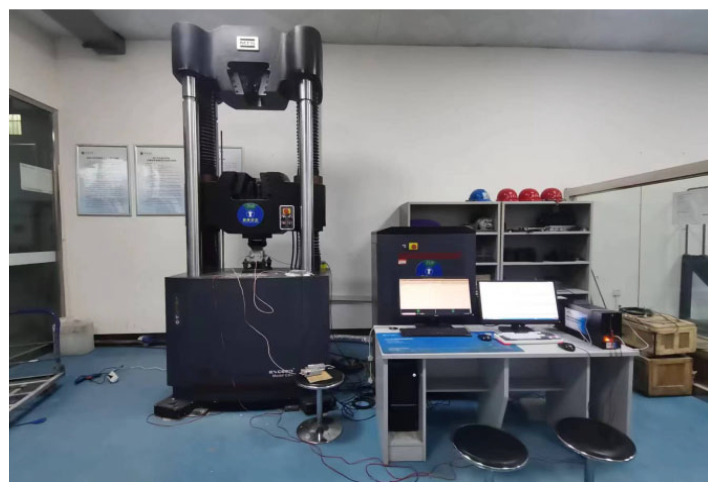
A 2000 kN microcomputer-controlled electro-hydraulic servo universal testing machine.

The testing was conducted using a 2000 kN microcomputer-controlled electro-hydraulic servo universal testing machine (Shanghai Hualong Testing Instrument Co., Ltd., Shanghai, China) at the Key Laboratory of Concrete Structure Safety and Durability in Shaanxi Province (Figure 3).

### 2.3. Determination of Optimal Fiber Content and Water Content in Specimens

Specimens were fabricated according to the mix proportions described in Section 2.1, with five different fiber contents (0.0%, 0.05%, 0.10%, 0.15%, and 0.20%). A total of 30 specimens were prepared, with six specimens for each fiber content level (three for uniaxial compression tests and three for splitting tensile tests). The uniaxial tensile and compressive strengths of the concrete specimens with different basalt fiber contents were measured to determine the optimal fiber content. The compressive and tensile test data are shown in Table 2. In the table, $\bar{\sigma }$_c_ represents the average compressive strength, *T*_c_ represents the increase rate of average compressive strength, $\bar{\sigma }$_t_ represents the average tensile strength, and *T*_t_ represents the increase rate of the average tensile strength.

As shown in Table 2, the compressive and tensile strengths of concrete first increase and then decrease with the rise in basalt fiber content. When the fiber content is 0.1%, the compressive strength of concrete reaches its maximum value of 36.90 MPa (an increase of 13.5% compared with the control group without fiber), and the tensile strength reaches its maximum value of 2.33 MPa (a rise of 15.9%).

### 2.4. Design of Scanning Electron Microscopy (SEM) Experiments

**Figure 4:**
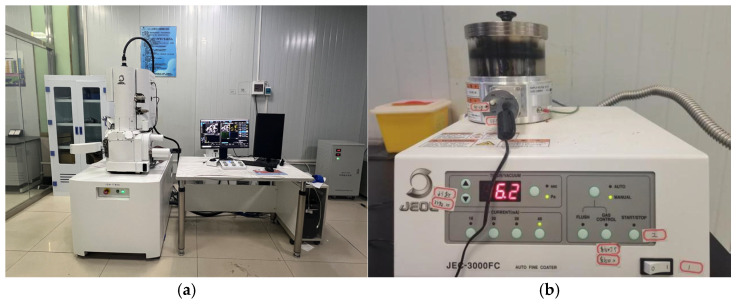
Scanning electron microscopy (SEM) testing equipment: (**a**) JEOL-JSM-IT800 thermal field emission scanning electron microscope; (**b**) JEC-300FC ion sputtering device.

Scanning electron microscopy (SEM) experiments were performed using the JEOL-JSM-IT800 thermal field emission scanning electron microscope (JEOL Ltd., Akishima, Tokyo, Japan) at the Key Laboratory of Organic and Polymeric Photoelectric Materials, Xijing University (Figure 4a). The detailed procedures are as follows: ① Sample Mounting: According to Reference [34], the samples were prepared into blocks with a diameter of no more than 10 mm. These samples were then affixed to the sample stage using conductive adhesive, ensuring that the analytical surface was parallel to the sample stage surface. ② Gold Coating: The samples were subsequently coated with gold using a JEC-300FC ion sputtering device (JEOL Ltd., Akishima, Japan) (Figure 4b). ③ Loading and Vacuuming: After placing the samples into the chamber and closing the lid, a vacuum is created. ④ Testing Initiation: Once the vacuum was established, the position of the sample stage was manually adjusted using the control lever to locate the region of interest on the sample surface. The contrast and brightness of the image were then fine-tuned, and the image was captured and saved. The testing procedure was repeated for each sample following the established protocol.

### 2.5. Design of Nuclear Magnetic Resonance (NMR) Experiments

**Figure 5:**
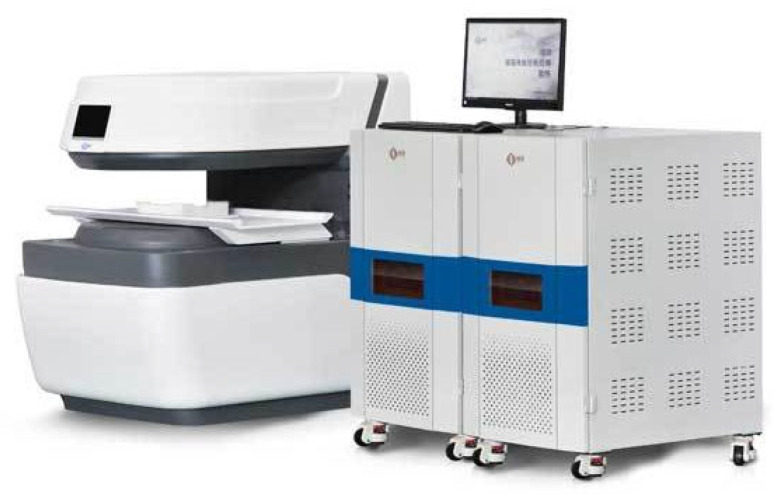
Low-field nuclear magnetic resonance measurement instrument, MacroMR12-150H-I.

**Figure 6:**
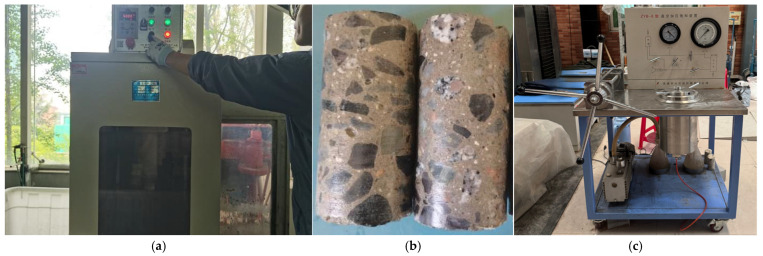
NMR testing procedures: (**a**) SC-300 concrete coring machine; (**b**) cored specimen; (**c**) ZYB-II vacuum-pressurized saturation apparatus.

The low-field nuclear magnetic resonance (NMR) measurement instrument used in this study is the MacroMR12-150H-I (Suzhou Niumag Analytical Instrument Co., Ltd., Suzhou, China), located in the Key Laboratory of Concrete Structure Safety and Durability in Shaanxi Province (Figure 5). The specific procedures are as follows: ① Core Extraction: The prepared specimens are placed into the SC-300 concrete coring machine (Shandong China Coal Industrial & Mining Supplies Group Co., Ltd., Jining, China) for core extraction (Figure 6a), resulting in a cylindrical concrete specimen with a diameter of 50 mm and a height of 100 mm (Figure 6b). ② Saturation Treatment: Step a: The cored specimen is placed into the pressurized chamber of the ZYB-II vacuum-pressurized saturation apparatus (Nantong Huaxing Petroleum Instruments Co., Ltd., Haian, China) (Figure 6c). The gases within the specimen’s pores and fissures are then evacuated until the vacuum gauge reading stabilizes, after which the specimen is left undisturbed for 8 h. Step b: The connection valve between the vacuum-pressurized saturation chamber and the vacuum pump is closed, while the valve connecting the vacuum-pressurized saturation chamber to the pressurized cylinder is opened. Step c: Water is introduced into the vacuum-pressurized saturation chamber, and the pressure is manually increased to 8 MPa, which is maintained for 24 h. ③ Testing Initiation: The specimen is placed into the NMR instrument, and the MacroMR12-150H-I NMR spectrometer (Suzhou Niumag Analytical Instrument Co., Ltd., Suzhou, China) and its associated control software are activated. ④ Parameter Settings: Upon entering the software, the 50 mm coil is selected, and the CPMG sequence is chosen. The O1, P1, and P2 parameters are calibrated based on standard samples, with the calibrated values being 242,790.3 Hz, 17 μs, and 34 μs, respectively. Other parameters are set as follows: SW = 250 kHz, PRG = 2, TW = 3000 ms, NS = 16, TE ≥ 6P2 at 0.25 ms, and NECH (number of echoes) = 12,000. ⑤ Conducting the Test: The test is carried out according to the set parameters, and the NMR data are collected and analyzed to evaluate the pore structure and hydration characteristics of the BFRC specimens.

### 2.6. Triaxial Compression Test Design

As shown in Section 2.3, the BFRC specimens with the optimal fiber content were successfully prepared. To further analyze the mechanical properties of BFRC specimens in a deep-water environment, the influence mechanisms of confining pressure and water content on the performance of BFRC were investigated through triaxial compression tests.

#### 2.6.1. Preparation of BFRC Specimens with Different Water Contents

**Figure 7:**
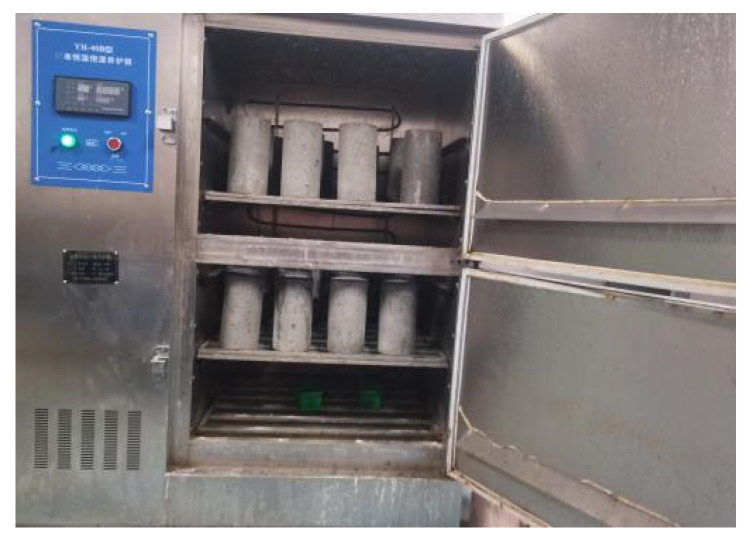
Specimen curing.

**Figure 8:**
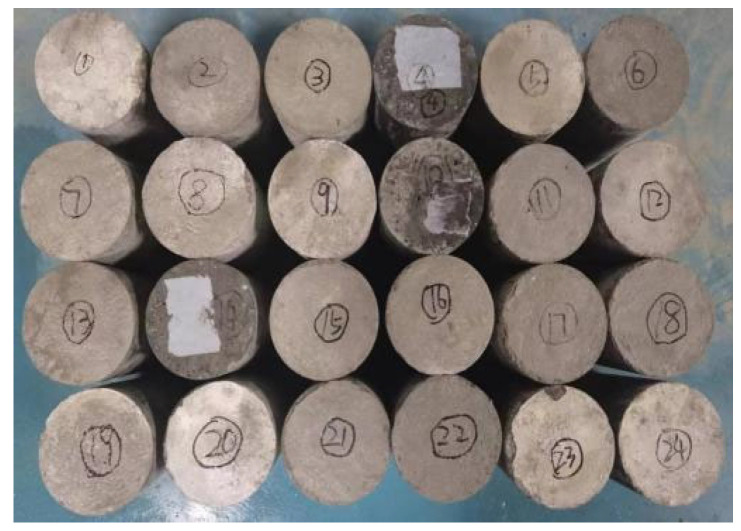
Specimen numbering.

**Figure 9:**
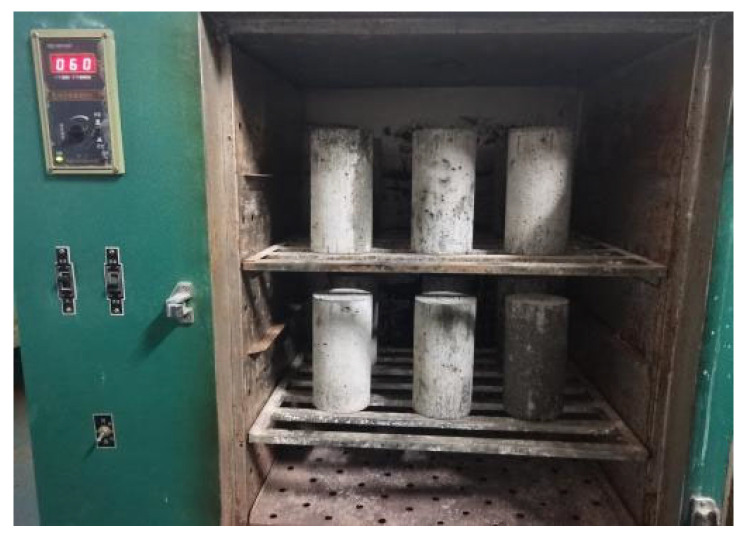
Specimen drying.

① Sample Preparation: A total of 30 cylindrical BFRC specimens with a diameter of 100 mm and a height of 200 mm were fabricated using a water-to-binder ratio of 0.10. The specimens were cast, allowed to rest for 24 h before demolding, and then cured in a standard curing chamber with a temperature of 20 ± 2 °C and a relative humidity of over 95% for 28 days (Figure 7). The specimens were subsequently numbered. To minimize the variability among the BFRC specimens, those with visible defects or non-uniform structures were excluded. Ultimately, 24 specimens were selected for further testing (Figure 8). These specimens were divided into three groups based on the magnitude of the confining pressure, with two specimens in each group. The specimens were labeled as BF-i-x, where BF denotes basalt fiber reinforced concrete, i represents the confining pressure level (in MPa), and x indicates the moisture content (in %). For each confining pressure level, two tests were conducted, and the result closest to the group average was selected as the representative outcome for analysis. ② Drying Specimens: The specimens were dried in a 101-1A type forced-draft electric heating constant temperature drying oven at a temperature of 60 °C (to avoid damage to the specimens) (Figure 9). The drying process continued until the mass of the specimen remained constant after 120 h, which was recorded as *m*_dry_. ③ Saturated Specimens: For saturation testing, the direct immersion method was used. The concrete specimens were placed in a water tank or trough to ensure that the concrete surface was completely submerged. The water quality was kept clean and at an appropriate temperature with regular inspection and replacement of the curing water. After 168 h of immersion, the water content remained unchanged, and the saturated water content was determined to be 4.16%, which was recorded as *m*_sat_. ④ Preparation of Specimens with Different Water Contents: The saturated BFRC specimens were placed in a drying oven and slowly dried at a temperature of 60 °C. The mass of the water-containing BFRC specimens was continuously monitored, and the water content of the BFRC specimens was calculated using the following formula: (*m* − *m*_dry_)/*m*_dry_, where *m* is the mass of the water-containing BFRC specimen and *m*_dry_ is the mass of the dry BFRC specimen. This process continued until BFRC specimens with water contents of 1% and 2% were prepared.

#### 2.6.2. Triaxial Compression Testing

**Figure 10:**
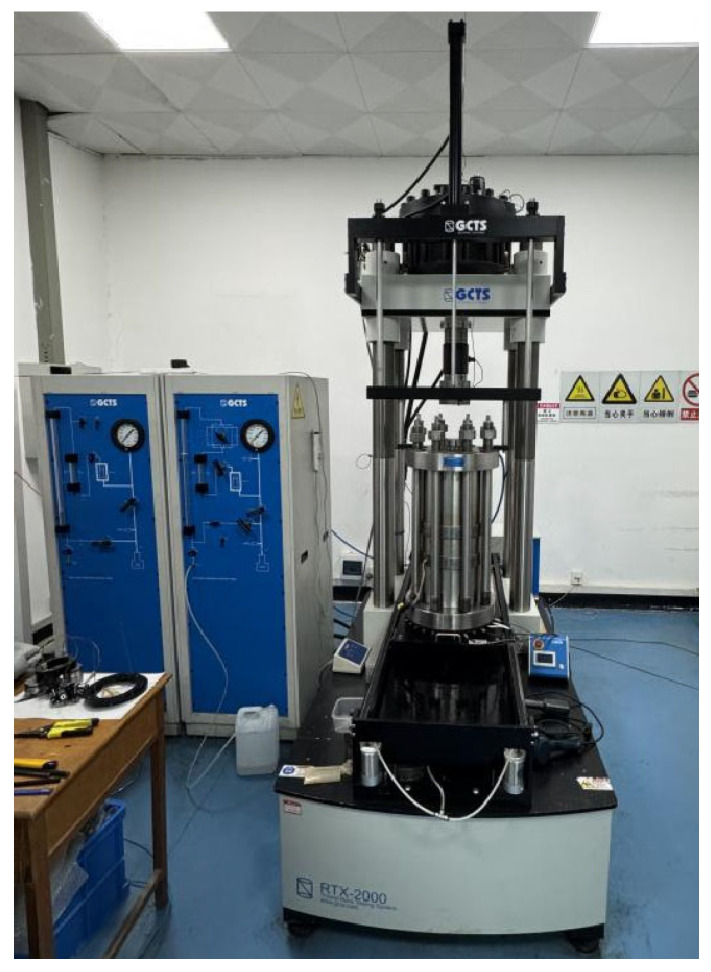
RTX-2000kN high-temperature and high-pressure electrohydraulic servo rock triaxial testing machine.

The experiments were conducted using the GCTS RTX-2000kN high-temperature and high-pressure electrohydraulic servo rock triaxial testing machine (GCTS Manufacturing Corp., Tempe, AZ, USA) from the Key Laboratory of Concrete Structure Safety and Durability in Shaanxi Province at Xijing University (Figure 10). The testing procedures followed Reference [35] and are described in detail below: ① Equipment Calibration: The triaxial testing machine was calibrated to ensure proper operation and accurate parameter settings, preparing for the subsequent experiments. ② Sample Preparation: The numbered samples were processed as follows. The surfaces of the samples were polished to achieve smoothness, and any residual cement mortar on the upper and lower surfaces was wiped clean with a towel to eliminate frictional forces that could affect the test results during loading. ③ Sample Installation: The processed samples were placed on the base plate. A heat shrink tube was fitted over the samples, and a heat gun was used to shrink the tube starting from the middle to ensure it tightly adhered to the sample surface. ④ Sensor Installation: Using a ruler, the total height of the spacer and sample was divided into three sections (74 mm, 157 mm, and 240 mm), and axial and circumferential sensors were installed. A level was used to ensure that the sensors were aligned horizontally. After installation, the sensor parameters were adjusted: the axial sensor was set to approximately −2.400 mm, and the circumferential sensor was set to approximately 5.700 mm. The base plate was then raised and retracted, and the pressure chamber was lowered, taking care not to press on the sensor wires. ⑤ Equipment Loading: Referring to the maximum underwater depth of the Seikan Tunnel in Japan, which is 449 m [2], and considering the influence of confining pressure in triaxial mechanical tests, the relationship between confining pressure and water depth is given by Equation (1):

*σ*_3_ = *ρ* × *g* × *H* × 10^−6^ (1)

In the formula, *σ*_3_ is the confining pressure (in MPa), *ρ* is the density of water (1000 kg/m^3^), *g* is the acceleration due to gravity (9.8 m/s^2^), and *H* is the water depth (in meters). The calculated confining pressure was approximately 4.4 MPa. Therefore, the confining pressure in this experiment was set to a range of 0–6 MPa with an interval of 3 MPa. The experimental parameters were entered into the system, and the loading strain rate was set to 0.05 mm/min [36]. A preloading of 1 MPa was applied and maintained for 5 min before the formal loading test began. If any issues arose during the test, the loading was restarted after the issues were resolved, continuing until the specimen failed. ⑥ Sample Unloading: The confining pressure was reduced to 0.1 MPa, and the ram was raised to create sufficient space to remove the ram. The fixing screws, rods, sensors, and heat shrink tubes were removed, and the failed specimen was taken out. Any residual debris on the equipment was cleaned, and the equipment was placed in an open area.

## 3. Mechanism of Strength Enhancement in Basalt Fiber-Reinforced Concrete (BFRC)

**Figure 11:**
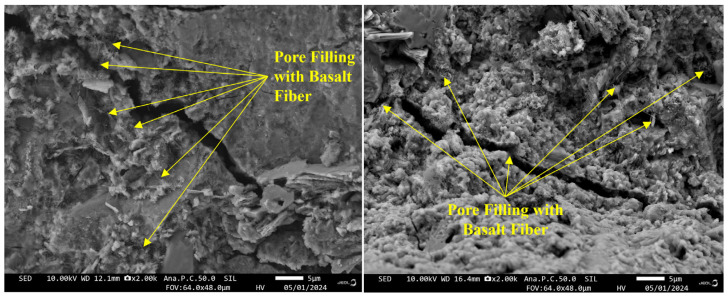
SEM Image of Basalt Fibers Filling Pores.

In Section 2, the optimal fiber content was determined through uniaxial compression and splitting tensile tests. It was observed that the compressive and tensile strengths of basalt fiber-reinforced concrete (BFRC) significantly outperformed those of plain concrete. This enhancement is primarily attributed to the random distribution characteristics of the fibers within the concrete matrix [37]. The reinforcing mechanism was further explored through microstructural analysis. Figure 11 presents the SEM image of basalt fibers filling the pores, which clearly shows that the addition of fibers significantly optimizes the internal pore structure of the concrete. On one hand, the incorporation of fibers can disrupt and reduce some pores, allowing cement paste to fill these voids. Some fibers can also fill larger pores, thereby further reducing the relative content of large pores. On the other hand, the bond between basalt fibers and the cement matrix is very strong. The randomly distributed fibers form a three-dimensional network structure among the concrete aggregates, which can effectively resist deformation stresses caused by temperature changes and drying shrinkage [38]. Specifically, under axial loading, the fibers exert lateral confining forces on the aggregates, forming a load-transferring fiber micromesh that further enhances the compressive strength of the concrete [39]. Moreover, the high elastic modulus and excellent tensile strength of basalt fibers enable them to disperse the load when the concrete is subjected to force, reducing the stress concentration. When cracks occur, the fibers can maintain the load-carrying capacity through bridging effects, transfer the load, and enhance the tensile properties while improving the interfacial transition zone performance, thereby increasing the splitting tensile strength [40,41]. However, when the fiber content exceeds 0.1%, the excessive addition of fibers hurts the compressive strength of concrete, causing it to decrease. The reason is that an excess of fibers tends to agglomerate within the concrete, forming localized fiber clusters. These clusters not only disrupt the homogeneity of the concrete but also reduce the effective bonding area between the fibers and the cementitious matrix, thereby diminishing the reinforcing effect of the fibers. More seriously, the fiber clusters act as stress concentration points, which further reduce the overall strength of the concrete [42]. This indicates that the fiber content needs to be precisely controlled to ensure its optimal reinforcing effect in concrete.

Furthermore, in the realm of pore structure research, Academician Wu Zhongwei [43] has placed significant emphasis on two critical factors: discrete porosity and pore size influence coefficient, which have provided valuable insights for the development of concrete materials towards a lightweight and high-strength direction. Based on the varying impacts of pore size on concrete strength, he classified the internal pores of concrete into four detailed categories: harmless pores (with a pore size less than 20 nm), slightly harmful pores (ranging from 20 to 50 nm), harmful pores (ranging from 50 to 200 nm), and highly harmful pores (exceeding 200 nm). Wu highlighted that increasing the number of pores smaller than 50 nm while reducing those larger than 100 nm can significantly enhance the performance of concrete [44]. This perspective aligns with the research of the renowned international scholar P. K. Mehta, who also noted that capillary pores larger than 50 nm have a more pronounced impact on the strength and permeability of concrete [45].

**Figure 12:**
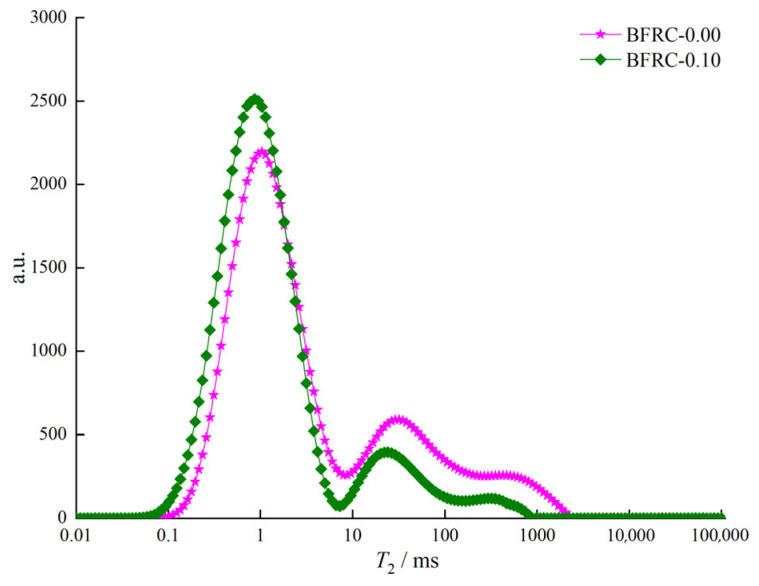
*T*_2_ Spectra of BFRC-0.00 and BFRC-0.10.

**Figure 13:**
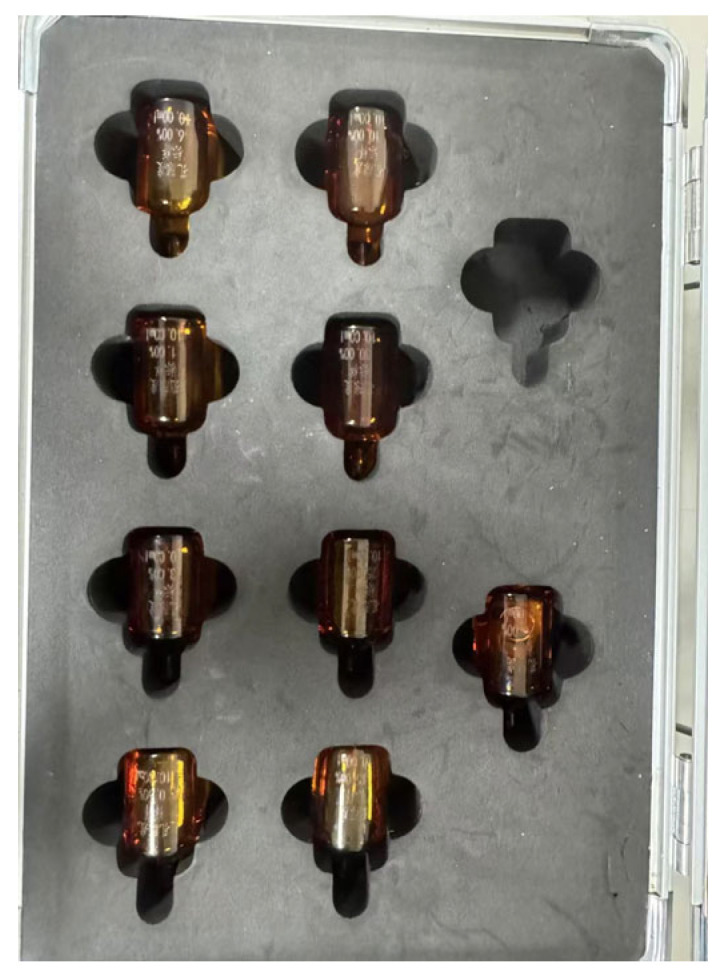
Standard samples with different porosities.

In the study of the pore structure of basalt fiber reinforced concrete (BFRC) using nuclear magnetic resonance (NMR) technology, pores are meticulously categorized into four levels: harmless pores, slightly harmful pores, harmful pores, and highly harmful pores. This classification is based on pore size, distribution, and their differential impacts on the mechanical properties and durability of concrete. Through NMR testing, the *T*_2_ spectra of BFRC-0.00 and BFRC-0.10 were obtained (Figure 12). Subsequently, the measured *T*_2_ spectra were substituted into the relationship between NMR-calculated porosity and the integral area of the *T*_2_ spectrum, derived from a standard sample with known porosity (Figure 13) (Equation (2)), to obtain the total porosity of the tested samples.

(2) $$\varphi_{NMR}=\left(\frac{S}{V}-3.65\right)/14.21$$

In the equation, *S* represents the integral area of the *T*_2_ spectrum (dimensionless), which reflects the total fluid relaxation characteristics in the sample; *V* refers to the volume of the sample (in cm^3^), used to standardize the porosity parameter; $\varphi_{NMR}$ is the porosity percentage calculated using low-field nuclear magnetic resonance (LF-NMR) technology (in %).

The pore structure of the specimens was further analyzed using nuclear magnetic resonance (NMR) technology. The NMR *T*_2_ spectrum can reflect the size and proportion of pores within the specimens. Specifically, there is a similar relationship between *T*_2_ relaxation time and pore size. The higher the signal amplitude of the *T*_2_ spectrum, the stronger the signal of water in the pores, indicating a larger pore volume. Through analysis of the *T*_2_ spectrum, it was found that the incorporation of an appropriate amount of basalt fiber (0.1%) can optimize the pore structure by reducing the number of large pores and increasing the proportion of small pores, thereby enhancing the density and mechanical properties of the concrete [46,47]. This can be expressed as follows:

*r* = 4 × *ρ*_2_ × *T*_2_ = *C* × *T*_2_ (3)

**Figure 14:**
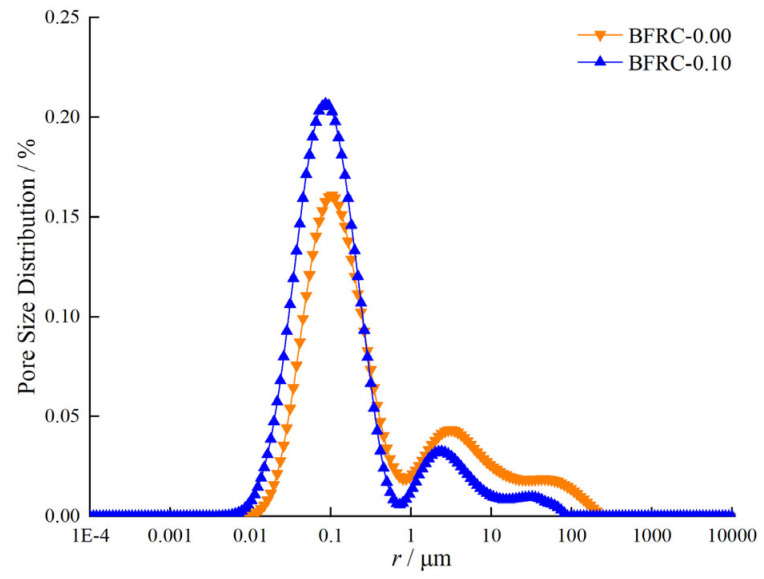
Pore radius distribution of BFRC-0.00 and BFRC-0.10.

In the equation, *r* is the pore radius inside the concrete (in nm); *C* is a constant conversion coefficient (in nm/ms); *ρ*_2_ is the surface relaxation rate, which describes the transverse relaxation intensity of the concrete material; and in the equation, *C* is 48 nm/ms [48,49]. This allows for the determination of the pore distribution and the porosity of different pore sizes (Figure 14).

Table 3 shows the total porosity and the porosity of each type of pore. It can be seen from Table 3 that the primary function of basalt fiber is to regulate the formation of the micro-porous structure within the concrete. This is achieved by altering the quantity and proportion of harmless pores and harmful pores. After an appropriate amount of fiber is added to the concrete, the proportion of harmless pores increases, while the proportions of slightly harmful pores, harmful pores, and highly harmful pores decrease somewhat. Specifically, the total porosity decreases from 7.48% to 7.43%, the proportion of harmless pores increases from 4.03% to 4.87%, the proportion of slightly harmful pores decreases from 1.06% to 0.76%, the proportion of harmful pores decreases from 0.72% to 0.38%, and the proportion of highly harmful pores decreases from 1.67% to 1.42%. As a result, the mechanical properties of the concrete are improved.

## 4. Analysis of Triaxial Compressive Mechanical Properties

**Figure 15:**
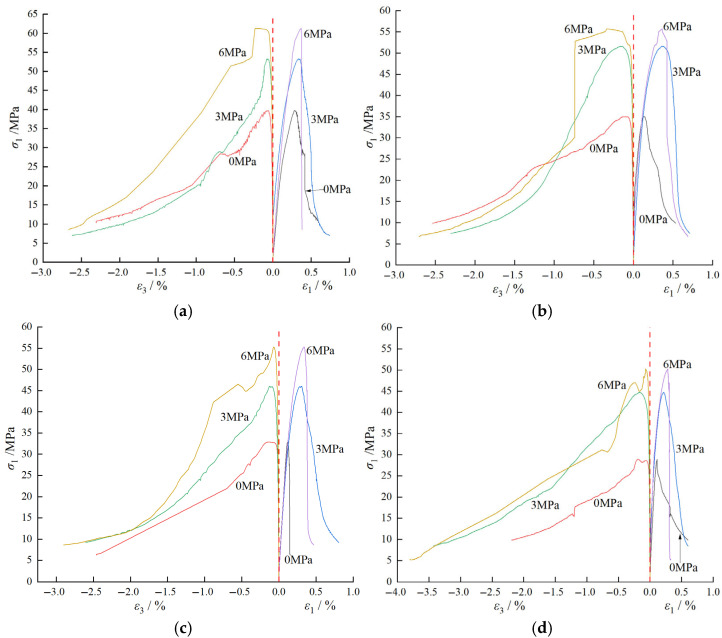
Full stress–strain curves of water-containing BFRC under different confining pressures: (**a**) water content of 0%; (**b**) water content of 1%; (**c**) water content of 2%; and (**d**) water content of 4.16%.

The full stress–strain curves of water-containing BFRC under different confining pressures are shown in Figure 15. In the figure, $\varepsilon_{1}$ and $\varepsilon_{3}$ represent the axial and radial strains, respectively, $\sigma_{1}$ is the axial pressure, and 0, 3, and 6 MPa are the confining pressures. The organized experimental data are presented in Table 4. In the table, BF is the abbreviation for BFRC, *H* is the height (mm), *D* is the diameter (mm), $\sigma_{3}$ is the confining pressure (MPa), $\sigma_{1m}$ is the axial peak stress (MPa), $\varepsilon_{1m}$ is the axial peak strain (%), and *E* is the elastic modulus (GPa); $\varepsilon_{vm}$ represents the peak volumetric strain (%).

### 4.1. Analysis of Deformation Characteristics

#### 4.1.1. Analysis of Stress–Strain Curve Characteristics

As shown in Figure 15, the triaxial stress–strain behavior of BFRC mainly includes four stages: initial crack compaction, elastic deformation, yielding, and failure, which are analyzed as follows: ① Initial Crack Compaction Stage: Due to the incorporation of basalt fibers, most of the pores in the concrete are filled, rendering the initial crack compaction stage less distinct. This stage is primarily characterized by minor deformation of the material at the beginning of loading, which prepares the material for the subsequent elastic deformation. ② Elastic Deformation Stage: In this stage, the stress–strain relationship of BFRC exhibits an approximately linear characteristic, indicating good elastic behavior. The stress is directly proportional to the strain, suggesting that the material can withstand significant loads without undergoing permanent deformation. ③ Yielding Stage: When the applied load exceeds the elastic limit of BFRC, the stress–strain curve begins to deviate from linearity, entering a nonlinear region. The axial stress growth slows down, while the strain increases rapidly. This indicates that internal cracks in the material start to propagate significantly, and the stiffness of the material gradually decreases. This stage is a critical transition from elastic to plastic behavior. ④ Failure Stage: With the continuous increase of axial load, BFRC reaches its strength limit. At this point, internal cracks interconnect to form distinct macroscopic fractures, which gradually expand. As the cracks propagate, the stress begins to decrease, signifying material failure. This stage is characterized by a significant drop in stress and an increase in deformation, representing the ultimate failure of the material.

Further analysis of the stress–strain curves in Figure 15 for the same water content under different confining pressures reveals that the confining pressure significantly influences the post-peak behavior of the specimens. When $\sigma_{3}$ is 0 MPa, the stress–strain curve drops steeply and irregularly after reaching $\sigma_{1m}$. However, when $\sigma_{3}$ is not 0 MPa, the confining pressure restricts the crack propagation during the softening phase, resulting in a smoother and more gradual softening process. With increasing confining pressure, specimens of the same strength exhibit significantly enhanced stiffness and peak stress, while the peak strain shifts to higher values, indicating greater ductility [50].

#### 4.1.2. Relationship Between Confining Pressure, Water Content, and Axial Peak Strain

**Figure 16:**
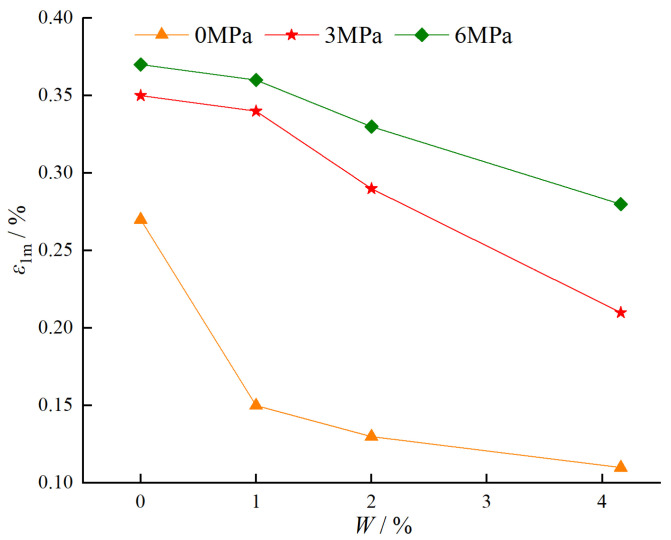
Variation of peak strain of BFRC specimens under different water contents and confining pressures.

Figure 16 illustrates the variation of peak strain of BFRC specimens under different water contents and confining pressures. As shown in Figure 16, when the confining pressure is constant, the peak strain of BFRC specimens decreases with increasing water content. Specifically, under a confining pressure of 0 MPa, as the water content increases from 0% to 4.16%, the peak strain decreases from 0.27% to 0.11%, with a reduction of 59.3%. Under a confining pressure of 3 MPa, as the water content increases from 0% to 4.16%, the peak strain decreases from 0.35% to 0.21%, with a reduction of 40.0%. Under a confining pressure of 6 MPa, as the water content increases from 0% to 4.16%, the peak strain decreases from 0.37% to 0.28%, with a reduction of 24.3%. The reasons for this phenomenon are as follows [24]: ① The erosive effect of water can cause the dissolution of the concrete material, thereby reducing its overall performance. ② The presence of water can reduce the interlocking and friction between aggregates, leading to softening of the specimen. Consequently, the peak stress and peak strain of the specimen are reduced. When the water content is constant, the peak strain of BFRC specimens increases with increasing confining pressure. Specifically, for a water content of 0%, the peak strain increases from 0.27% at 0 MPa confining pressure to 0.35% at 3 MPa and further to 0.37% at 6 MPa. The increases in peak strain are 29.6% and 37.0%, respectively. For a water content of 1%, the peak strain increases from 0.15% at 0 MPa confining pressure to 0.34% at 3 MPa, and further to 0.36% at 6 MPa. The increases in peak strain are 126.7% and 140.0%, respectively. For a water content of 2%, the peak strain increases from 0.13% at 0 MPa confining pressure to 0.29% at 3 MPa and further to 0.33% at 6 MPa. The increases in peak strain are 123.1% and 153.8%, respectively. For a water content of 4.16% (saturated), the peak strain increases from 0.11% at 0 MPa confining pressure to 0.21% at 3 MPa and further to 0.28% at 6 MPa. The increases in peak strain are 90.9% and 154.5%, respectively. This occurs because the confining pressure limits the lateral expansion of the specimen, allowing it to better utilize the material’s load-bearing capacity under axial compression, thereby increasing the peak strain.

#### 4.1.3. Relationship Between Confining Pressure, Water Content, and Elastic Modulus

**Figure 17:**
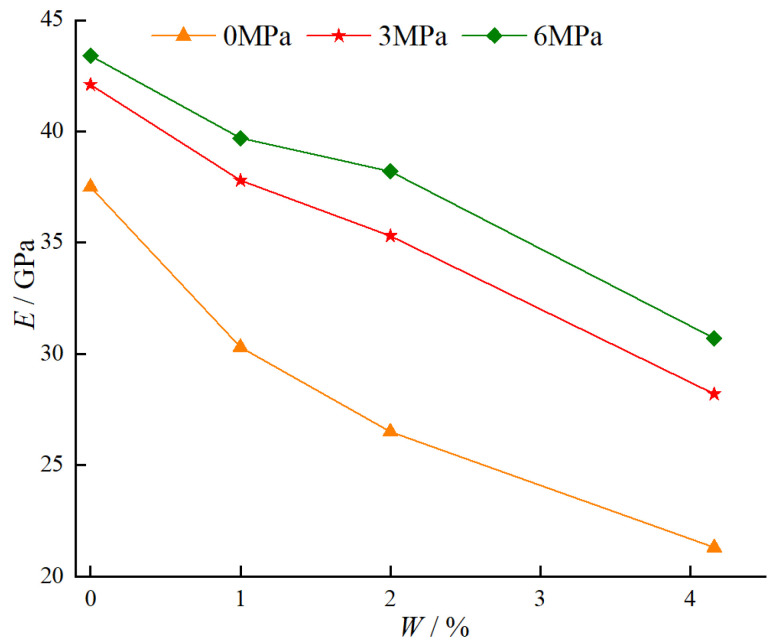
Variation of elastic modulus of BFRC specimens under different water contents and confining pressures.

Figure 17 illustrates the variation of the elastic modulus of BFRC specimens under different water contents and confining pressures. As shown in Figure 17, when the confining pressure is constant, the elastic modulus decreases with increasing water content. Specifically, under a confining pressure of 0 MPa, as the water content increases from 0% to 4.16%, the elastic modulus decreases from 37.5 GPa to 21.3 GPa, with a reduction of 43.2%. Under a confining pressure of 3 MPa, as the water content increases from 0% to 4.16%, the elastic modulus decreases from 42.1 GPa to 28.2 GPa, with a reduction of 33.0%. Under a confining pressure of 6 MPa, as the water content increases from 0% to 4.16%, the elastic modulus decreases from 43.4 GPa to 30.7 GPa, with a reduction of 29.3%. The reason for this trend is that an increase in water content weakens the inter-particle bonding and friction within the material, thereby reducing its overall stiffness. When the water content is constant, the elastic modulus increases with increasing confining pressure. Specifically, for a water content of 0%, the elastic modulus increases from 37.5 GPa at 0 MPa confining pressure to 43.4 GPa at 6 MPa confining pressure, with an increase of 15.7%. For a water content of 1%, the elastic modulus increases from 30.3 GPa at 0 MPa confining pressure to 39.7 GPa at 6 MPa confining pressure, with an increase of 31.0%. For a water content of 2%, the elastic modulus increases from 26.5 GPa at 0 MPa confining pressure to 38.2 GPa at 6 MPa confining pressure, with an increase of 44.2%. For a water content of 4.16%, the elastic modulus increases from 21.3 GPa at 0 MPa confining pressure to 30.7 GPa at 6 MPa confining pressure, with an increase of 43.7%. The reason for this trend is that the confining pressure causes the particles and fibers within the specimen to be compressed more uniformly, enhancing the overall stiffness of the specimen and thereby increasing the elastic modulus.

#### 4.1.4. Relationship Between Confining Pressure, Water Content, and Volumetric Strain

**Figure 18:**
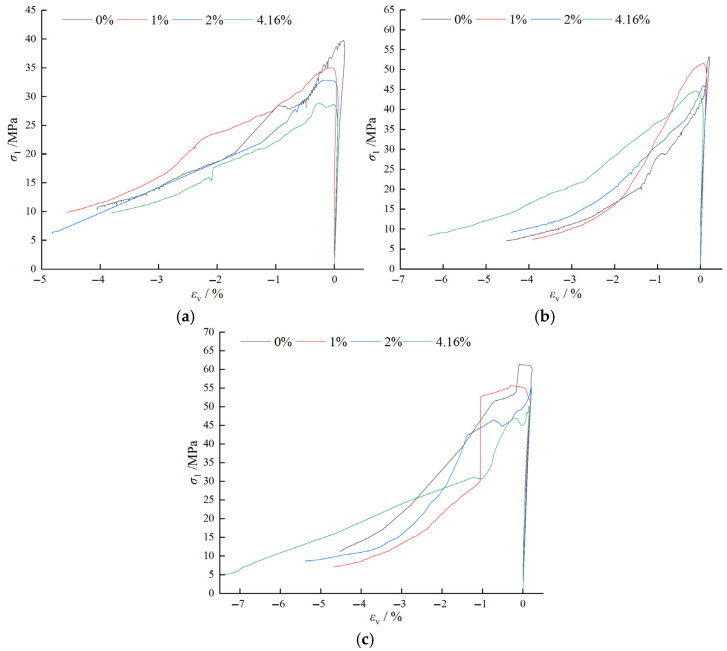
Triaxial compression full stress-volumetric strain curves of BFRC under different confining pressures: (**a**) confining pressure of 0 MPa; (**b**) confining pressure of 3 MPa; and (**c**) confining pressure of 6 MPa.

Figure 18 illustrates the full stress–volumetric strain curves of BFRC specimens under different water contents and confining pressures. The volumetric strain is calculated using the formula $\varepsilon_{v}$ = $\varepsilon_{1}$ + ${2\varepsilon }_{3}$ [51]. As shown in Figure 18, with the increase of confining pressure, the elastic stage of BFRC specimens with four different water contents becomes steeper, and the curves overlap better, indicating that the lateral confinement under high confining pressure has a more uniform effect on the deformation characteristics of the material, thereby reducing the influence of differences in water content. Secondly, when $\sigma_{3}=0\;MPa$, the inflection points of BFRC specimens with all four water contents are located near the peak points. When $\sigma_{3}=3\;MPa$, the inflection points of BFRC specimens with water contents of 0%, 1%, and 2% are still near the peak points, but for the saturated specimens, the inflection points shift to the left, indicating an earlier onset of volumetric expansion. This suggests that a high water content may increase the pore water pressure within the material, causing it to exhibit significant plastic deformation at lower stresses and thus enter the volumetric expansion stage earlier. When $\sigma_{3}=6\;MPa$, the inflection points of BFRC specimens are near the peak points (except for the specimen with 1% water content), indicating that under high confining pressure, the lateral confinement has a significant effect on the deformation characteristics of the material, reducing the influence of water content on the location of the inflection points.

**Figure 19:**
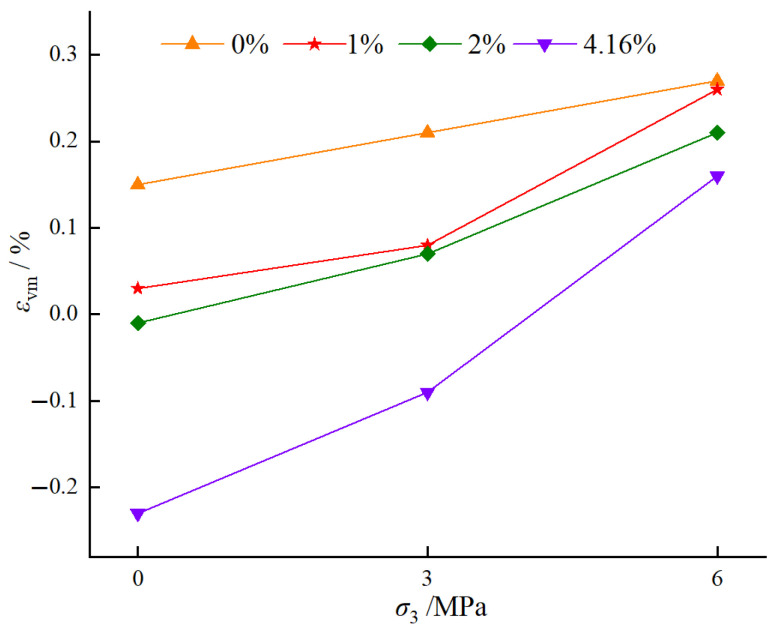
Variation of peak volumetric strain of BFRC specimens under different water contents and confining pressures.

Figure 19 illustrates the variation of peak volumetric strain of BFRC specimens under different water contents and confining pressures. As shown in Figure 19: ① Effect of Confining Pressure on Peak Volumetric Strain: When the water content is constant, the peak volumetric strain ($\varepsilon_{vm}$) increases with increasing confining pressure. Specifically, for a water content of 0%, $\varepsilon_{vm}$ increases from 0.15% at 0 MPa confining pressure to 0.27% at 6 MPa, an increase of 80.0%. For a water content of 1%, $\varepsilon_{vm}$ increases from 0.21% at 0 MPa confining pressure to 0.26% at 6 MPa, an increase of 23.8%. For a water content of 2%, $\varepsilon_{vm}$ increases from 0.03% at 0 MPa confining pressure to 0.21% at 6 MPa, an increase of 600.0%. For a water content of 4.16%, $\varepsilon_{vm}$ increases from −0.01% at 0 MPa confining pressure to 0.16% at 6 MPa, an increase of 1700.0%. This is because the confining pressure provides lateral restraint, limiting the lateral expansion of the specimen during axial compression. This allows the material to sustain greater volumetric strain before failure. ② Effect of Water Content on Peak Volumetric Strain: When the confining pressure is constant, $\varepsilon_{vm}$ decreases with increasing water content. Specifically, at 0 MPa confining pressure, as the water content increases from 0% to 4.16%, $\varepsilon_{vm}$ decreases from 0.15% to −0.01%, a reduction of 106.7%. At 3 MPa confining pressure, as the water content increases from 0% to 4.16%, $\varepsilon_{vm}$ decreases from 0.21% to 0.07%, a reduction of 66.7%. At 6 MPa confining pressure, as the water content increases from 0% to 4.16%, $\varepsilon_{vm}$ decreases from 0.27% to 0.21%, a reduction of 22.2%. This is because water reduces the bonding strength within the material, thereby weakening its overall strength. As a result, the material fails at lower stress levels, leading to a decrease in $\varepsilon_{vm}$. ③ Mitigating Effect of Confining Pressure on the Influence of Water Content: As the confining pressure increases, the differences in $\varepsilon_{vm}$ among the four water content levels of BFRC specimens become smaller. This is because, under high confining pressure, the lateral restraint has a dominant effect on the deformation characteristics of the material, overshadowing the influence of water content differences. Consequently, the peak volumetric strains of specimens with different water contents tend to converge.

### 4.2. Analysis of Failure and Strength Characteristics

#### 4.2.1. Analysis of Failure Characteristics

**Figure 20:**
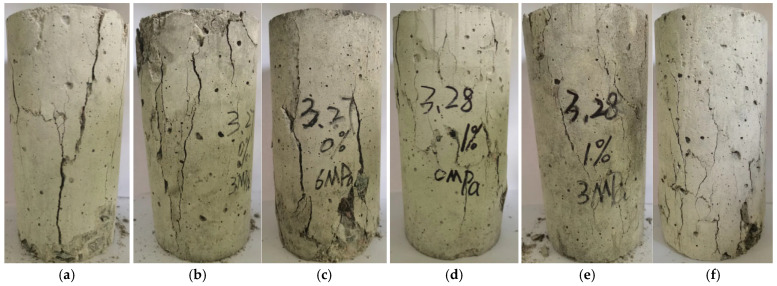

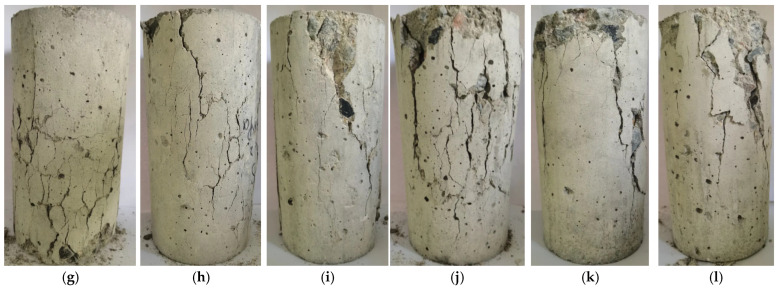
Failure characteristics of BFRC specimens under different water contents and confining pressures: (**a**) BF-0-0; (**b**) BF-3-0; (**c**) BF-6-0; (**d**) BF-0-1; (**e**) BF-3-1; (**f**) BF-6-1; (**g**) BF-0-2; (**h**) BF-3-2; (**i**) BF-6-2; (**j**) BF-0-S; (**k**) BF-3-S; and (**l**) BF-6-S.

Figure 20 shows the failure characteristics of BFRC specimens under different water contents and confining pressures. The failure characteristics of BFRC specimens exhibit distinct stages of change with increasing confining pressure and water content. Specifically: ① At a 0 MPa Confining Pressure: For all four water content levels, the failure mode of BFRC specimens is primarily longitudinal splitting. At 0% water content, the failure is characterized by a single, nearly vertical through-crack. At 1% water content, the crack becomes finer and more numerous. As the water content increases further, the crack width increases, and the number of cracks multiplies, with spalling occurring in areas of dense crack networks. The reason is that at 0% water content, the internal pore structure of the specimen is relatively compact, resulting in higher material strength. Under axial compression, the specimen mainly exhibits a through-going longitudinal crack. However, as the water content increases, the pores within the specimen are filled with water, which weakens the internal bonding strength of the material and reduces its strength. Consequently, the specimen is more prone to multiple fine cracks under loading. With further increases in water content, the crack width and number increase, and the spalling in crack-dense areas is due to localized stress concentration and reduced material strength. ② At a Non-zero Confining Pressure: The lateral confining pressure restricts the transverse strain of the specimen, causing vertical cracks to gradually evolve into diagonal cracks. The failure mode transitions from vertical splitting to diagonal shear failure. Specifically: a. When the water content is constant: As the confining pressure increases, the failure becomes more severe, characterized by more spalling, more and wider cracks, and more pronounced bulging in the middle of the specimen. This is because the increased confining pressure provides stronger lateral restraint, limiting the transverse deformation of the specimen. Under this constraint, the internal stress distribution of the specimen becomes more complex, with more stress concentration zones and higher stress levels. When the stress exceeds the material’s strength limit, the specimen is more likely to fail, and the failure form is more violent. Therefore, the specimen exhibits more spalling, an increased number of cracks, and wider crack widths. Additionally, due to stress concentration and complex stress distribution, the bulging in the middle of the specimen becomes more evident. b. When the confining pressure is constant: The failure degree of water-containing specimens is generally less severe than that of dry specimens. This is because the internal water in the water-containing specimens acts as a lubricant, reducing the internal frictional resistance during loading and thus subjecting the specimen to smaller forces. However, as the water content increases, the structural stability of the specimen gradually decreases. The increase in water content alters the internal pore structure of the specimen, reducing its strength and toughness. Consequently, the failure degree becomes more severe, characterized by more spalling and wider cracks.

#### 4.2.2. Analysis of Strength Characteristics

**Figure 21:**
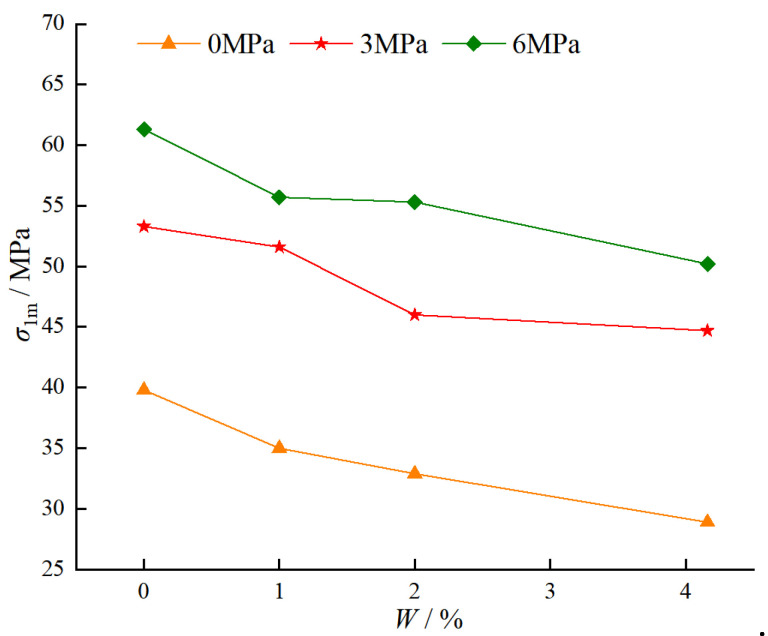
Variation of peak strength of BFRC specimens under different water contents and confining pressures.

Figure 21 presents the variation of peak strength of BFRC specimens under different water contents and confining pressures. As shown in Figure 21, ① when the confining pressure is constant, the peak strength decreases with increasing water content. Specifically, under a confining pressure of 0 MPa, as the water content increases from 0% to 4.16%, the peak stress decreases from 39.8 MPa to 28.9 MPa, with a reduction of 27.4%. Under a confining pressure of 3 MPa, as the water content increases from 0% to 4.16%, the peak stress decreases from 53.3 MPa to 44.7 MPa, with a reduction of 16.1%. Under a confining pressure of 6 MPa, as the water content increases from 0% to 4.16%, the peak stress decreases from 61.3 MPa to 50.2 MPa, with a reduction of 18.1%. The reason for this trend is that an increase in water content weakens the interfacial bond between the basalt fibers and the cement matrix, reducing the efficiency of stress transfer. Additionally, water filling the pores changes the pore structure and increases internal defects. These factors collectively lead to the easier formation and propagation of microcracks under loading, thereby reducing the peak strength. ② When the water content is constant, the peak strength increases with increasing confining pressure. Specifically, for a water content of 0%, the peak stress increases from 39.8 MPa at 0 MPa confining pressure to 61.3 MPa at 6 MPa confining pressure, with an increase of 54.0%. For a water content of 1%, the peak stress increases from 35.0 MPa at 0 MPa confining pressure to 55.7 MPa at 6 MPa confining pressure, with an increase of 60.3%. For a water content of 2%, the peak stress increases from 32.9 MPa at 0 MPa confining pressure to 55.3 MPa at 6 MPa confining pressure, with an increase of 68.1%. For a water content of 4.16%, the peak stress increases from 28.9 MPa at 0 MPa confining pressure to 50.2 MPa at 6 MPa confining pressure, with an increase of 73.0%. This arises as the confining pressure constrains the specimen’s lateral deformation, leading to a more uniform stress distribution and inhibiting the formation and propagation of microcracks. Additionally, the presence of confining pressure optimizes the reinforcing effect of the fibers, allowing them to more effectively transfer stress to the matrix, thereby enhancing the overall strength of the material.

## 5. Numerical Simulation of Triaxial Compression of BFRC Under Different Water Content Levels

The Concrete Damaged Plasticity (CDP) model in ABAQUS, which comprehensively considers both the stiffness degradation and irreversible deformation of the material, is capable of effectively simulating the nonlinear characteristics of concrete. It has been widely applied in the analysis of various concrete structures [52].

### 5.1. Model Design

**Figure 22:**
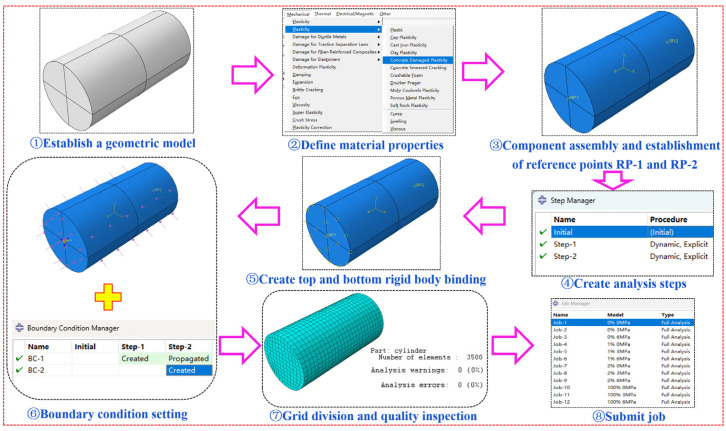
Modeling steps in ABAQUS.

The mechanical properties of water-containing BFRC under triaxial stress were simulated and analyzed using the Concrete Damaged Plasticity (CDP) model in the Abaqus 2024 software. The modeling steps are shown in Figure 22 and are as follows: ① Geometry Model Establishment: A cylindrical specimen with a diameter of 100 mm and a height of 200 mm was created using the geometry module in ABAQUS. ② Material Property Definition: A new material was defined through the material manager, and the CDP model was selected. For the elastic part, the modulus of elasticity (*E*) was set differently for BFRC specimens with different water contents based on triaxial compression test data, while the Poisson’s ratio (*ν*) was set to 0.2 [53]. The density (*ρ*) was determined by weighing the specimens and was set to 2500 kg/m^3^. For the plasticity part, the plastic parameters were referenced from [54], and the specific elastoplastic parameters are listed in Table 5. For the damage part, the compressive and tensile stress–strain curves of concrete were obtained by referencing [53]. ③ Assembly and Reference Point Creation: The specimen was assembled, and two reference points (RP-1 and RP-2) were created to represent the top and bottom surfaces. ④ Analysis Step Creation: Two analysis steps were created, with the first step used to apply confining pressure and the second step to apply axial pressure. A dynamic, explicit analysis step was selected to ensure convergence. ⑤ Rigid Body Binding for Top and Bottom Surfaces: Rigid body binding was applied to allow RP-1 and RP-2 to represent the top and bottom surfaces, respectively. ⑥ Boundary Condition Setup: Stress boundary conditions were applied using the Load module in ABAQUS, with confining pressures of 0, 3, and 6 MPa applied to all surfaces except the bottom surface in Step 1. Displacement boundary conditions were then applied: all translational degrees of freedom were constrained on the bottom surface (RP-2) in Step 1 to simulate a fixed base, and a displacement of −10 mm was applied in the Z-direction on the top surface (RP-1) in Step 2 to induce specimen failure. ⑦ Meshing: The model was meshed using C3D8 elements, and the mesh quality was checked to ensure no errors or warnings. ⑧ Job Submission: The job was submitted for numerical simulation, and the results were analyzed.

### 5.2. Model Validation

**Figure 23:**
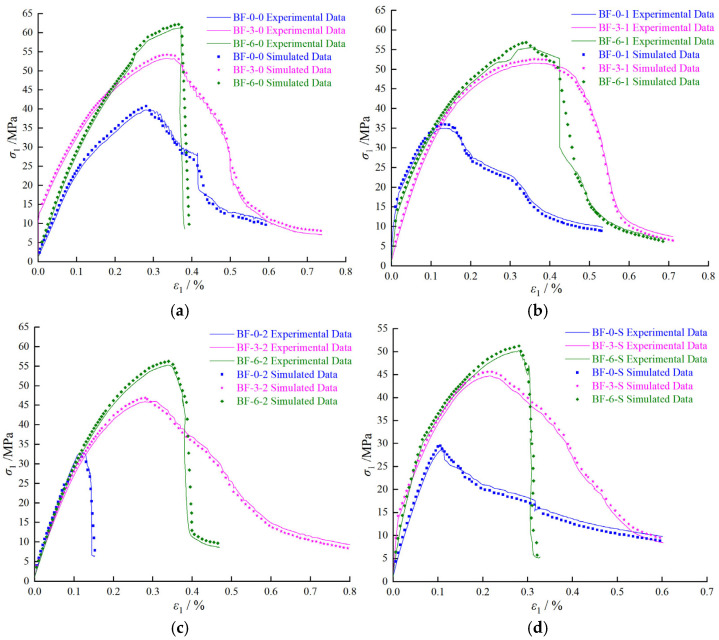
The comparison between the triaxial compression test results and numerical simulation results for water-containing BFRC specimens: (**a**) water content of 0%; (**b**) water content of 1%; (**c**) water content of 2%; and (**d**) water content of 4.16%.

Figure 23 shows the comparison between the triaxial compression test results and numerical simulation results for water-containing BFRC specimens. As can be seen from Figure 23, the trends of the simulation results are generally consistent with those of the experimental results. Specifically, with the increase of confining pressure, the peak stress of BFRC significantly increases, indicating the strengthening effect of confining pressure on the material strength. Meanwhile, the increase in water content leads to a decrease in both peak stress and peak strain, suggesting that water content hurts the mechanical properties of BFRC. Moreover, at a confining pressure of 0 MPa, the influence of water content on material properties is the most pronounced, while the effect of water content on mechanical properties weakens with the increase of confining pressure. These conclusions are consistent with those obtained from the experiments, fully demonstrating that the model can effectively predict the failure process of water-containing BFRC specimens under triaxial compression conditions.

## 6. Conclusions

This study systematically investigated the mechanical properties of optimally water-containing basalt fiber-reinforced concrete (BFRC) under triaxial stress conditions through comprehensive experiments and analysis. The main conclusions are as follows:

(1) Optimal Fiber Content: Through uniaxial compression and splitting tensile tests, the optimal fiber content for BFRC was determined to be 0.1%. At this dosage, the compressive strength of the concrete increased by 13.5% compared to the control group without fibers, reaching 36.90 MPa. The tensile strength also improved by 15.9%, reaching 2.33 MPa. This indicates that an appropriate amount of basalt fiber can significantly enhance the mechanical properties of concrete.
(2) Microstructural Optimization: Basalt fibers optimize the pore structure and form a network-like three-dimensional structure, significantly enhancing concrete strength. However, exceeding the optimal dosage of 0.1% leads to a decrease in strength, highlighting the need for precise control of fiber content. The pore size distribution was also optimized, with the total porosity decreasing from 7.48% to 7.43%, the proportion of harmless pores increasing from 4.03% to 4.87%, and the proportion of harmful pores decreasing from 1.67% to 1.42%. This microstructural optimization makes the concrete more compact, reduces stress concentration, and thereby improves macroscopic mechanical properties.
(3) Stress–Strain Curve Characteristics: BFRC specimens under triaxial stress–strain conditions primarily experience four stages: initial crack compaction, elastic deformation, yielding, and failure. The initial crack compaction stage is not obvious. During the elastic deformation stage, the stress–strain relationship is linear. In the yielding stage, the stress–strain curve deviates from linearity, entering a nonlinear region. During the failure stage, the stress gradually decreases, indicating material failure. Increasing confining pressure restricts crack propagation during the softening phase, resulting in a smoother softening process.
(4) Mechanical Properties: Peak strain significantly increases with increasing confining pressure. For example, at 0 MPa confining pressure and 0% water content, the peak strain is 0.27%, while at 6 MPa confining pressure, it increases to 0.37%. However, increasing water content reduces peak strain. For instance, at 0 MPa confining pressure, increasing the water content from 0% to 4.16% reduces the peak strain from 0.27% to 0.11%. Elastic modulus is also significantly affected by confining pressure and water content. It increases with confining pressure but decreases with increasing water content. For example, at 0 MPa confining pressure, increasing the water content from 0% to 4.16% reduces the elastic modulus from 37.5 GPa to 21.3 GPa. Volumetric strain shows similar trends, with peak volumetric strain increasing with confining pressure and decreasing with water content. Peak strength significantly increases with confining pressure but decreases with increasing water content, indicating that the enhancing effect of confining pressure on BFRC mechanical properties is significantly superior to the negative impact of water content.
(5) Failure Characteristics: The failure characteristics of BFRC change in stages with variations in confining pressure and water content. Under zero confining pressure, specimens primarily exhibit longitudinal splitting failure. As confining pressure increases, the failure mode gradually transitions to diagonal shear failure. Increasing confining pressure leads to more severe failure, characterized by more and wider cracks and more pronounced bulging in the middle of the specimen. In contrast, water-containing specimens exhibit less severe failure, but as water content increases, the failure degree gradually worsens, with wider and more numerous cracks. This indicates that confining pressure significantly affects failure characteristics, while water content initially mitigates the failure degree but eventually has a negative impact as it increases further.
(6) Numerical modeling: A triaxial compression model for BFRC under various confining pressures and moisture contents was developed using the CDP model in ABAQUS. The simulation results align well with experimental data, confirming the model’s accuracy and applicability. This provides a reliable numerical tool for predicting the mechanical behavior of BFRC in complex stress environments and for supporting the design and analysis of engineering structures.

The findings of this study provide significant theoretical support for the application of high-performance concrete in marine and structural engineering. In marine environments, concrete structures are often subjected to complex triaxial stress states and high humidity conditions. The appropriate addition of basalt fiber can significantly enhance the mechanical properties and durability of concrete. In engineering projects such as cross-sea tunnels and port wharfs, the use of BFRC with optimized fiber content can effectively improve the compressive and tensile strengths of the structures, reduce the generation and propagation of cracks, and thereby extend the service life of the structures. Moreover, by understanding the influence mechanisms of confining pressure and water content on the mechanical properties of BFRC, more precise parameters can be provided for engineering design, optimizing the durability and stability of structures.

## Figures and Tables

**Table 1:** Mix design of BFRC.

Number	Fiber Content /%	Cement /kg/m^3^	Coarse Aggregate /kg/m^3^	Fine Aggregate /kg/m^3^	Admixture /kg/m^3^	Additives /kg/m^3^	Water /kg/m^3^
BFRC-0.00	0.00	280	950	900	100	9	115
BFRC-0.05	0.05	280	950	900	100	9	115
BFRC-0.10	0.10	280	950	900	100	9	115
BFRC-0.15	0.15	280	950	900	100	9	115
BFRC-0.20	0.20	280	950	900	100	9	115

**Table 2:** Compressive and tensile strength test results.

Number	$\bar{\sigma }$_c_ /MPa	*T*_c_ /%	$\bar{\sigma }$_t_ /MPa	*T*_t_ /%
BFRC-0.00	32.50	—	2.05	—
BFRC-0.05	35.24	8.4	2.11	5.1
BFRC-0.10	36.90	13.5	2.33	15.9
BFRC-0.15	35.52	9.2	2.09	6.7
BFRC-0.20	32.70	0.6	2.06	2.5

**Table 3:** The total porosity and the porosity of each grade of pores.

Number	Total Porosity/%	The Porosity of Each Grade of Pores/%			
		Harmless Pores	Slightly Harmful Pores	Harmful Pores	Highly Harmful Pores
BFRC-0.00	7.48	4.03	1.06	0.72	1.67
BFRC-0.10	7.43	4.87	0.76	0.38	1.42

**Table 4:** Specimen parameters and triaxial compression test results.

Number	*H*	*D*	$\sigma_{1m}$	$\varepsilon_{1m}$	E	$\varepsilon_{vm}$
	/mm	/mm	/MPa	/10^−2^	/GPa	/10^−2^
BF-0-0	199.97	99.99	39.8	0.27	37.5	0.15
BF-3-0	199.96	99.95	53.3	0.35	42.1	0.21
BF-6-0	200.03	99.98	61.3	0.37	43.4	0.27
BF-0-1	199.94	99.96	35.0	0.15	30.3	0.03
BF-3-1	199.98	98.99	51.6	0.34	37.8	0.08
BF-6-1	199.96	98.97	55.7	0.36	39.7	0.26
BF-0-2	199.89	100.02	32.9	0.13	26.5	−0.01
BF-3-2	199.87	98.56	46.0	0.29	35.3	0.07
BF-6-2	199.78	97.02	55.3	0.33	38.2	0.21
BF-0-S	199.72	97.82	28.9	0.11	21.3	−0.23
BF-3-S	200.01	100.01	44.7	0.21	28.2	−0.09
BF-6-S	199.69	100.04	50.2	0.28	30.7	0.16

**Table 5:** Modeling parameters for BFRC with different water content levels.

Water Content	*ρ* /kg/m^3^	*E* /GPa	*v*	Dilatancy Angle	Eccentricity	Biaxial to Uniaxial Compressive Strength Ratio	Hardening Coefficient	Viscosity Coefficient
0%	2500	37.5	0.2	30	0.1	1.16	0.6667	0.05
1%	2500	30.3	0.2	30	0.1	1.16	0.6667	0.05
2%	2500	26.5	0.2	30	0.1	1.16	0.6667	0.05
4.16%	2500	21.3	0.2	30	0.1	1.16	0.6667	0.05

## Data Availability

The original contributions presented in this study are included in the article. Further inquiries can be directed to the corresponding authors.
